# Current and Future Distribution of Striped Hyena in Nepal

**DOI:** 10.1002/ece3.72167

**Published:** 2025-09-17

**Authors:** Surya Devkota, Bashu Dev Baral, Sandeep Regmi, Bishnu Prasad Bhattarai, Shivish Bhandari, Hem Bahadur Katuwal, Basudha Rawal, Jerrold L. Belant, Rachana Shah, Chiranjibi Prasad Pokheral, Naresh Subedi, Hari Prasad Sharma

**Affiliations:** ^1^ Central Department of Zoology, Institute of Science and Technology Tribhuvan University Kathmandu Nepal; ^2^ Southeast Asia Biodiversity Research Institute, Chinese Academy of Sciences & Center for Integrative Conservation, Xishuangbanna Tropical Botanical Garden Chinese Academy of Sciences Mengla Yunnan China; ^3^ The Himalayan Conservancy Kathmandu Nepal; ^4^ Nepal Zoological Society Kathmandu Nepal; ^5^ Natural Science Society Kathmandu Nepal; ^6^ Department of Fisheries and Wildlife Michigan State University East Lansing Michigan USA; ^7^ National Trust for Nature Conservation Lalitpur Nepal

**Keywords:** conservation, distribution, habitat, hyena, scavenger

## Abstract

The striped hyena *Hyena hyena* , a facultative scavenger inhabiting undulating forest and grassland ecosystems, has experienced significant shifts in its distribution due to human activities, such as land use and climate change. Despite these shifts, the extent and direction of these changes have not been explored to inform site‐specific management and conservation strategies. We assessed current and future potential suitable habitats for striped hyena using species distribution modeling (SDM), considering bioclimatic and land use variables. We obtained striped hyena occurrence data throughout Nepal from field surveys and existing literature. Using ensemble modeling, we predicted 17.01% (25,199 km^2^) of Nepal's land as suitable habitat for striped hyenas, with 23.15% of suitable habitat occurring inside protected areas. Key environmental factors influencing habitat suitability included temperature, precipitation, and proximity to water sources. We also predicted substantial declines in suitable habitats by 2070, with reductions of 74% and 79.3% under SSP1‐2.6 and SSP5‐8.5 scenarios. Based on these findings, we highlight the importance of extending conservation efforts beyond protected areas to safeguard current and future habitats of striped hyenas.

## Graphical abstract

The hyenas suitable habitat will be decreased in the future if the current scenarios continue.

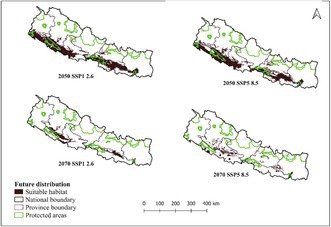

## 1 Introduction

The striped hyena *Hyena hyena* is a facultative scavenger (Bhandari et al. 2020; Panda et al. 2022; Singh et al. 2010) that occurs in habitats ranging from arid and semi‐arid to grassland, forest, and urban areas (Bhandari, Bhusal, et al. 2021; Hadad et al. 2023; Kruuk 1976; Wagner 2006). The global distribution of striped hyena includes North and East Africa, Middle Eastern and Central Asia, and the Indian subcontinent, including Nepal (AbiSaid and Dloniak 2015; Hofer and Mills 1998). In Nepal, it occurs in western and central lowlands from elevations of 100–2136 m (Suwal and Verheugt 1995; Bhandari and Bhusal 2017; Jnawali et al. 2011). The global estimated population of this species is less than 10,000 individuals (Abi‐Said and Abi‐Said 2007), whereas in Nepal it is less than 100 individuals (Jnawali et al. 2011). The population has declined due to retaliatory killing, illegal trade, road mortality, accidental trapping, and habitat loss (AbiSaid and Dloniak 2015; Alam et al. 2015). In addition, loss of natural habitats including forests and grassland areas threatens striped hyena persistence in Nepal (Bhandari et al. 2020), exacerbated by anthropogenic disturbances including tree cutting, livestock grazing, and forest fires (DFRS 2014).

Climate change and landscape alterations can negatively impact ecosystems, influencing species interactions within and across trophic levels (Jin et al. 2024; Ullah et al. 2018). Climate change impacts species distributions, potentially altering species abundance and distributions, increasing homogenization of community composition across regions, and changing community structures (Antão et al. 2022; Bhattacharjee et al. 2017; Thuiller et al. 2006; Urban 2015). However, the magnitude and direction of these range shifts vary among species and taxonomic groups (Rubenstein et al. 2023). Furthermore, increased urbanization and other human‐induced land use changes have dramatically altered the Earth's surface since the 1900s (Devkota et al. 2023). Compared to other guilds, carnivores are especially sensitive to climate change because of their unique biological traits, typically smaller populations, lower reproduction rates, and large home ranges (Leão et al. 2023).

Species distribution modeling (SDM) has commonly been used to understand species‐environment relationships and forecast spatial distributions of species (Naimi and Araújo 2016). This is important in conservation science as these models provide systematic approaches to characterize and forecast geographical distributions of species based on environmental conditions (Elith and Leathwick 2009). Distribution models also are useful to predict future range under climate change and inform conservation efforts, ensuring the long‐term survival of species (Acharya et al. 2023; Elith and Leathwick 2009). Ensemble models that integrate multiple distribution model types have broad application (Araújo and New 2007) and are considered superior to single distribution models for predicting climate projections of threatened species (Araújo and New 2007).

Among large carnivores in Nepal, striped hyenas are one of the least studied despite their importance in ecosystem function and conservation (Bhandari and Bhusal 2017). There is limited information on the current and future potential distribution of striped hyenas. However, a previous study (Bhandari et al. 2022) explored the impact of climate change on hyenas. While the study (Bhandari et al. 2022) examines the potential impact of climate change on hyenas using a single SDM approach, our study employs an ensemble modeling framework that integrates multiple algorithms to improve prediction accuracy and reduce model uncertainty. Furthermore, we incorporate updated occurrence records and higher‐resolution environmental variables, including the land use variables at both present and future scenarios, allowing us to estimate future climate scenarios more robustly, an aspect limited in the previous study. This comprehensive approach offers a more nuanced understanding of habitat shifts and potential climate refugia for hyenas, providing valuable insights for long‐term conservation planning beyond those presented in the earlier publication.

In this study, we use ensemble modeling to estimate the current potential habitat of striped hyenas in Nepal and to assess how future climate and land use change may alter their predicted distribution. By comparing current and forecasted habitat suitability, our goal is to provide insights that can inform national conservation strategies and help prioritize actions to mitigate the potential impacts of land use and climate change on the distribution of striped hyenas.

## 2 Materials and Methods

### 2.1 Study Area

**FIGURE 1:**
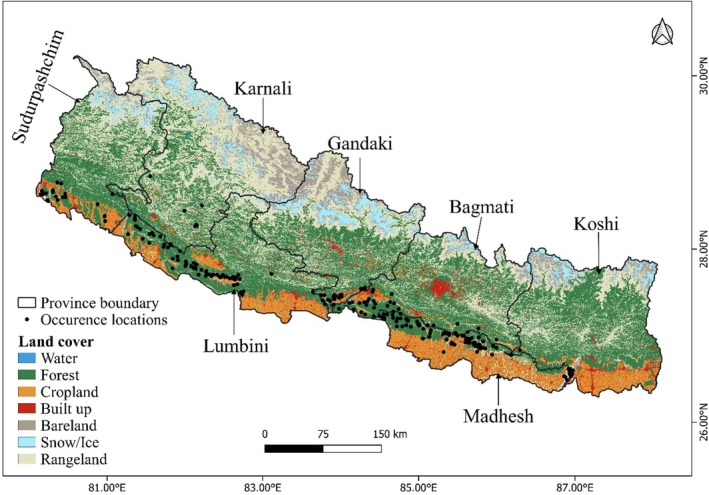
Confirmed occurrence locations of striped hyena in Nepal.

Nepal (26°22′–30°27′ N 80°04′–88°12′ E; Figure 1) is in Central Asia between China and India and comprises 147,516 km^2^. Elevations range from 60 to 8848 m (Bhattacharjee et al. 2017) grouped into five elevation zones, Tarai, Siwalik, Mid‐Hills, Mid‐Mountains, and High Mountain (Carson et al. 1986), and five climate zones, that is, tropical and subtropical (< 1200 m), temperate (1200–2400 m), cold (> 2400–3600 m), subarctic (3600–4400 m), and arctic (> 4400 m) (Li and Deng 2017). Nepal receives average annual rainfall of around 1530 mm in which 80%–90% occurs during the monsoon (July–September) (Bhattacharjee et al. 2017; Shrestha 2000). Precipitation increases from west to east (Pokharel et al. 2020), resulting in higher biodiversity in eastern Nepal (Paudel et al. 2011).

### 2.2 Research Permission

We received the research permission from the Department of Forest and Soil Conservation (Permission ID: 476) and the Department of National Parks and Wildlife Conservation (Permission ID: 1165). Data for this research was collected without animal handling.

### 2.3 Methods

We used both the primary and secondary data for distribution modeling of striped hyenas. We used remote camera data collected during December 2022–May 2023 in Madhesh Province, Nepal (Sharma et al. 2024), and from December 2024 to April 2025 in Lumbini Province and Shuklaphanta National Park, Nepal. Also, we extracted locations provided in previously published sources (Khanal et al. 2017; Bhandari et al. 2020, Bhandari, Youlatos, et al. 2021; Bhandari et al. 2022; Lamichhane et al. 2021), and from the Global Biodiversity Information Facilities (GBIF). We used only verified presence points from GBIF.

A total of 23 presence points were obtained from the camera trap survey, 159 from unpublished sources, 13 from published sources, and 91 from GBIF. In total, 277 occurrence points for striped hyenas were identified. However, these points were checked for spatial filtering to confirm a single point within a 1 × 1 km to avoid sampling biases and spatial auto‐correlation (Boria et al. 2014). Finally, a total of 228 occurrence points were used for modeling.

We retrieved 19 bioclimatic layers (bio1‐bio19) during 1970–2000 from WorldClim (Version 2.1) (https://www.worldclim.org/ data/bioclim.html) with a spatial resolution of 30 arc‐seconds (~1 km^2^) (Fick and Hijmans 2017) (Table S1). As land use land cover patterns also impact the distribution of large carnivores (Mkonyi et al. 2018), we downloaded a cellular automaton downscaling‐based 1 km global land use dataset (2010–2100) from Figshare (Li et al. 2016) at 1‐km^2^ resolution. We downloaded land use variables such as forest, grassland, farmland, barren land, shrub cover, impervious surfaces, and water and later transformed these variables into raster distance files using the proximity raster in QGIS (Version 3.22.13).

We used the Coupled Model Intercomparison Project 6 (CMIP6) to determine the variables to retrieve from WorldClim 2.1 (Fick and Hijmans 2017). Furthermore, we downloaded bioclimatic data for 13 Global Climate Models (GCMs): ACCESS‐CM2, CMCC‐ESM2, EC‐EARTH3‐Veg, FIO‐ESM2, GFDL‐ESM4, GISS‐E2‐1‐G, HadGEM3‐GC31‐LL, INM‐CM5‐0, IPSL‐CM6A‐LR, MIROC6, MPI‐ESM1‐2‐HR, MRI‐ESM2‐0, UKESM1‐0‐LL, available in the CMIP6 for the years 2050 and 2070. We averaged values from these 13 models for analysis to improve reliability (Murphy et al. 2004; Shrestha et al. 2022). Four Shared Socioeconomic Pathways (SSPs) were available for each model to consider the potential effects of climate change on species. We used SSP1 2.6 and SSP5 8.5 for modeling to represent extremes whereby SSP1 2.6 represents a scenario with low emissions and a 2°C warmer world, and SSP5 8.5 represents a scenario with high emissions and a 5.1°C warmer world (Baral et al. 2023; Katuwal et al. 2023).

We extracted values from all bioclimatic and land use variables using the Point sampling tools plugins in QGIS (Version 3.22.13). We conducted Spearman's pairwise correlations of variables in R Programming (R Core Team 2023) to exclude highly correlated variables (Figure S1) and excluded variables with high correlation (|*r*| > 0.7) from analyses. After correlation analysis, we retained 10 variables, that is, bio1, bio2, bio3, bio12, bio14, bio15, bio17, and distances to forest, impervious (Impervious surfaces are hard surfaces that prevent water from seeping into the ground.), and water for modeling (Table S1).

We created an ensemble model using six algorithms: generalized linear model (GLM), recursive partitioning and regression trees (RPART), generalized additive model (GAM), multivariate additive regression splines (MARS), random forest (RF) and boosted regression tree (BRT) using the “sdm” package (Naimi and Araújo 2016) in program R (R Core Team 2023). We created 5000 random background points as pseudo‐absence points required for analysis, then separated presence and background points into training (70%) and testing datasets (30%). To ensure that there was less probability of bias toward species absence sites, we used an elevational limit of 2500 m above sea level, which is similar to the elevation the species was previously observed in Nepal (AbiSaid and Dloniak 2015; Bhandari and Bhusal 2017; Lamichhane et al. 2021). We conducted 10 replications (Katuwal et al. 2023; Malla et al. 2023) to increase accuracy and robustness (Araújo and Guisan 2006) for two subsampling and bootstrap processes using the six algorithms, for a total of 120 individual models. We used the weighted mean approach to enhance the accuracy of the final ensemble models (Naimi and Araújo 2016).

We evaluated model performance using area under the curve (AUC) of the receiver operating characteristics (Phillips et al. 2006) and True Skill Statistics (TSS) (Allouche et al. 2006). We considered AUC scores from 0.6 to 0.7 as poor, from 0.7 to 0.8 as fair, from 0.8 to 0.9 as good, and > 0.9 as excellent (Phillips et al. 2006). For TSS scores, values < 0.4 indicate poor model discrimination and a value of 1 indicates perfect discrimination (Allouche et al. 2006). We then used a maximum sensitivity plus specificity logistic threshold to transform projected probabilities into presence or absence (Liu et al. 2011).

## 3 Results

### 3.1 Model Performance and Predictor Variable Contribution

The model predicted striped hyena distribution with high accuracy (AUC = 0.92 ± 0.02, TSS = 0.80 ± 0.04). Overall, RF was the best‐performing model to predict striped hyena distribution, followed by BRT, with RPART performing poorest.

**FIGURE 2:**
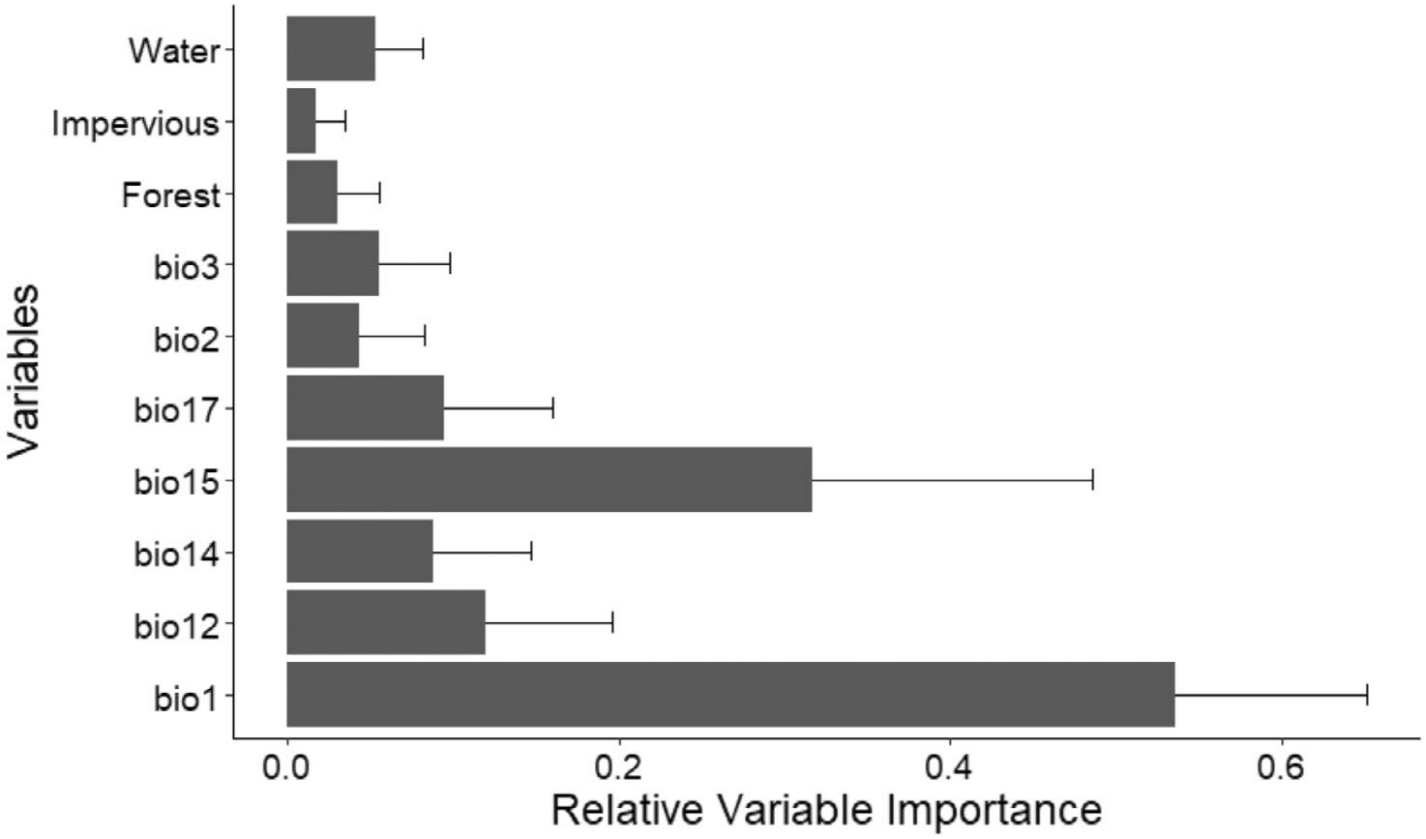
Relative Variable Importance for the distribution of striped hyena in Nepal. Annual mean temperature (bio1), annual precipitation (bio12), precipitation of driest month (bio14), precipitation seasonality (coefficient of variation) (bio15), precipitation of driest quarter (bio17), mean diurnal range (mean of monthly (max temp—min temp)) (bio2), isothermality (bio2/bio7) (×100) (bio3).

**FIGURE 3:**
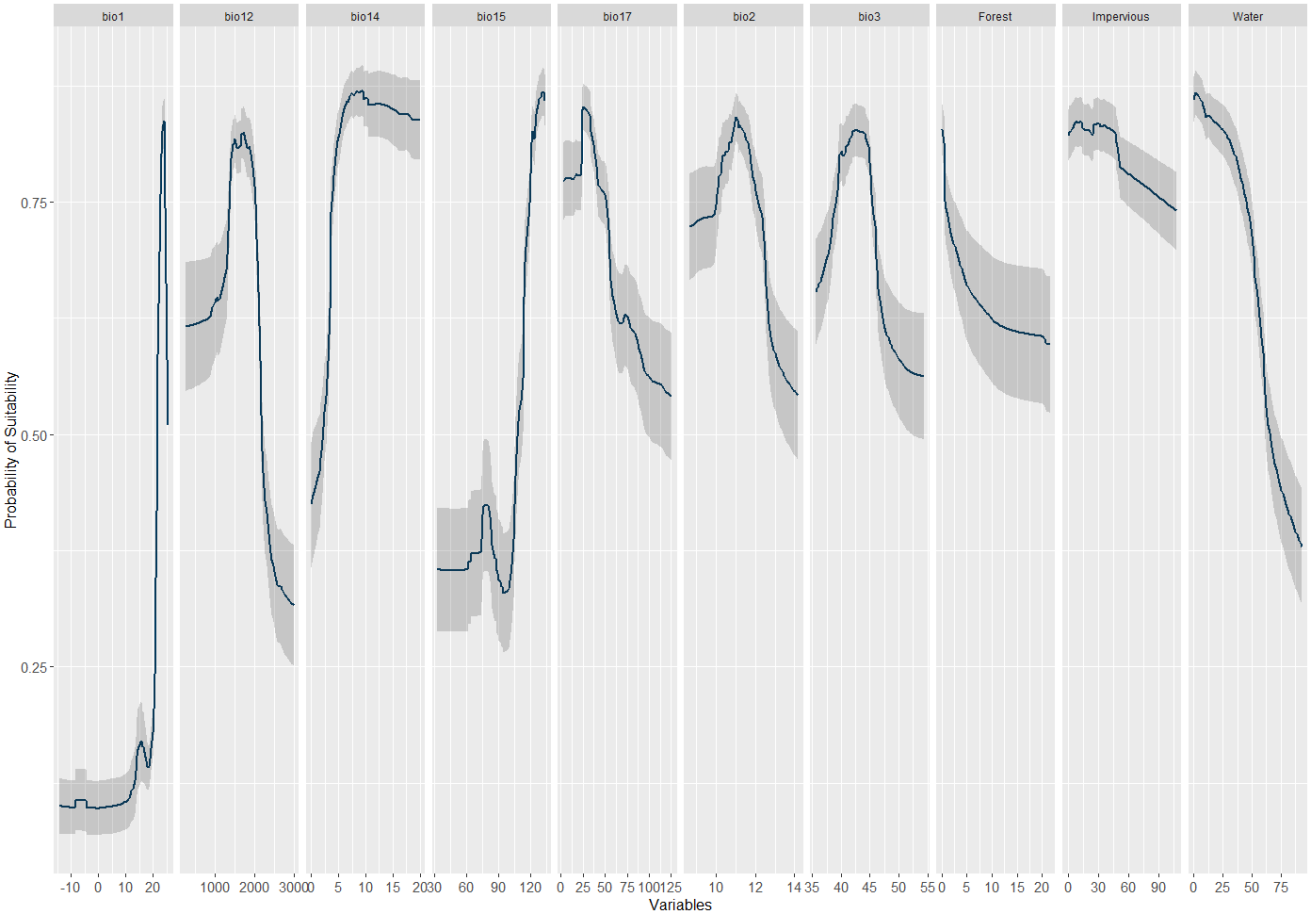
Response curves for the distribution of striped hyena in Nepal. Annual mean temperature (bio1), annual precipitation (bio12), precipitation of driest month (bio14), precipitation seasonality (coefficient of variation) (bio15), precipitation of driest quarter (bio17), mean diurnal range (mean of monthly (max temp—min temp)) (bio2), isothermality (bio2/bio7) (×100) (bio3).

The most influential variables for predicting striped hyena distribution were annual mean temperature (bio1), followed by precipitation seasonality (coefficient of variation) (bio15), annual precipitation (bio12), precipitation of driest month (bio14), and distance to water (Figure 2). The probability of suitable habitat increased with increasing precipitation seasonality, annual mean temperature, precipitation of driest month (bio14), and distance to forest. In contrast, the probability of habitat decreased with increasing mean diurnal range (mean of monthly (max. temp–min temp)) (bio2), isothermality (bio3), and distance to water (Figure 3).

### 3.2 Current Predicted Distribution

**FIGURE 4:**
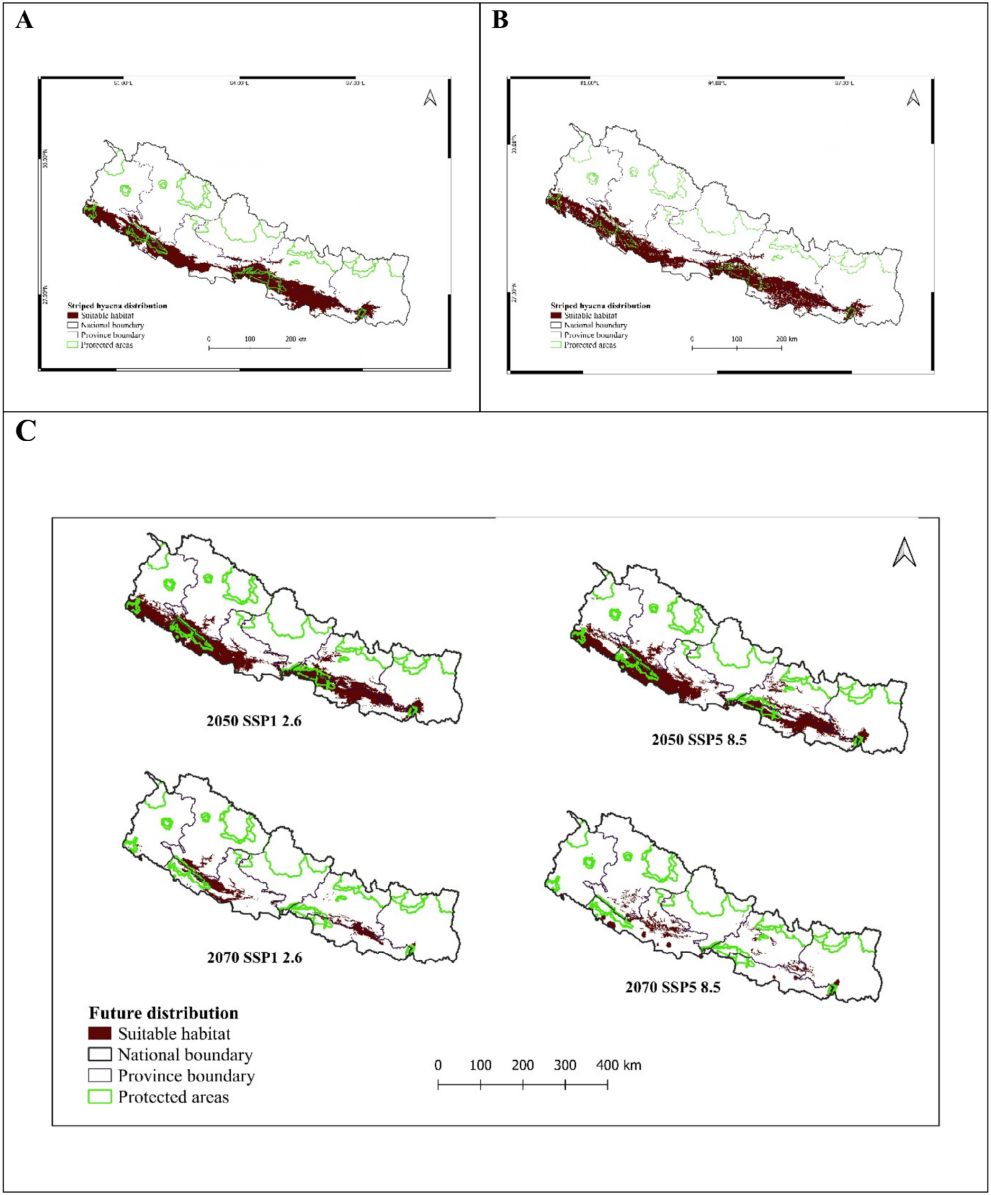
Current and future distribution of striped hyena in Nepal under bioclimatic and land use land change: (A) current distribution, (B) current distribution excluding settlement area, (C) future distribution under different scenarios.

Our ensemble model identified 17.01% (25,199 km^2^) of Nepal as suitable habitat for striped hyenas (Table 1, Figure 4A). Only 23.15% (3690.93 km^2^) of suitable habitat occurred within protected areas. However, only 15% (23,317 km^2^) remained suitable when excluding the suitable area that overlaps with settlement (Figures 4B and S2). In the context of province level, Madhesh province (65.78% of its total landmass) contained the most potential habitat for striped hyena, followed by Lumbini (39.26%), Bagmati (20.65%), and Sudurpashchim (17.71%) provinces.

**TABLE 1:** Predicted area (%) of striped hyena distribution in Nepal based on current and future climate and land use change scenarios.

Climatic scenario	Predicted habitat (%)	Loss from current (%)
Current	17.01	0
2050 SSP1‐2.6	17.71	−4.1
2050 SSP5‐8.5	16.49	3.05
2070 SSP1‐2.6	4.37	74.3
2070 SSP5‐8.5	3.52	79.3

*Note:* SSP1‐2.6 and SSP5‐8.5 refers to the first and last scenarios.

### 3.3 Future Predicted Distribution of Striped Hyena

Both climatic scenarios (SSP1‐2.6 and SSP5‐8.5) and land cover/land use change impacted striped hyena potential distribution. There were declines in distribution in both future scenarios (2050 and 2070). In 2050, a slight increase (4.1%) in suitable habitat was observed under SSP1‐2.6, whereas it declined by 3.05% under the SSP5‐8.5 scenario (Table 1, Figure 4). By 2070, both SSP1‐2.6 and SSP5‐8.5 scenarios predicted declines of 74% and 79.3%, respectively, with a slightly upward elevation shift in the hyena potential distribution (Table 1, Figures 4 and S3).

## 4 Discussion

We identified current and future potential habitat of striped hyenas in Nepal, and revealed probable declines in future potential habitat due to climate and land use change (AbiSaid and Dloniak 2015; Bhandari et al. 2022; Neupane et al. 2021). Potential habitat increased with increasing annual mean temperature (up to 24°C); striped hyenas are thermally sensitive and their physiology is influenced by ambient temperature (McNab 2000; Panda et al. 2022). While higher temperatures could expand their habitat range, they may also lead to increased water and energy requirements, potentially altering their foraging behavior and habitat selection (Godde et al. 2021). Potential habitat of striped hyenas also increased with precipitation seasonality between 105 and 115 mm and decreased with precipitation of driest month between 10 and 20 mm. Precipitation seasonality leads to increased availability of food resources as precipitation leads to fluctuation in vegetation which directly affects herbivore populations that are a primary prey of striped hyenas (Alam and Khan 2015). Moreover, an increase in precipitation of driest month reduces the competition between striped hyenas and other species for water resources and forage (Kluever et al. 2017). This relationship is further supported by observed declines in potential habitat distant from open water, previously demonstrated to play an important role in striped hyena habitat selection (Alam et al. 2014; Wagner 2006). Though striped hyenas can persist in arid and semi‐arid environments, these variables collectively suggest a positive association between striped hyenas and warmer, wetter climates (Alam and Khan 2015; Dadashi‐Jourdehi et al. 2020; Shamoon and Shapira 2019; Singh et al. 2010).

We predicted only 12% of Nepal's land as suitable habitat for striped hyena, mostly concentrated in the central and western lowlands (Madhesh and Lumbini provinces). These lowlands offer diverse habitats including grasslands, scrublands, and forests (Paudel et al. 2011) which provide suitable habitat for many ungulates and small mammal species that are primary prey of hyenas (Alam and Khan 2015). Only 20% of potential habitat occurred in protected areas, despite hyenas selecting for areas near human settlements to forage (Abi‐Said and Abi‐Said 2007; Almasieh et al. 2022; Bhandari, et al. 2020; Panda et al. 2022), including at refuse dumps, livestock farms, and at carcasses (Alam et al. 2014; Bhandari, Bhusal, et al. 2021; Singh et al. 2010). Most protected areas in the lowland region of Nepal provide potential habitat for striped hyenas. Similarly, outside protected areas, Sarlahi, Rautahat, Mahottari, and Dang districts contained most potential habitat for striped hyenas. These areas could provide more foraging opportunities for hyenas due to more human settlements and agricultural lands (Bhandari, Bhusal, et al. 2021). These districts also contain fragmented forest patches, which could facilitate hyena avoidance of humans.

Projection models suggest a gradual decline in the potential distribution of striped hyenas under land use change and both climate scenarios (SSP1‐2.6 and SSP5‐8.5). This underscores the vulnerability of striped hyenas to these impacts and highlights the need for conservation measures to mitigate their effects. The impact of land use and land cover change, along with climate change, is well known to result in reductions in the distribution of species (Jin et al. 2024; Liu et al. 2016), and climate change can exacerbate impacts due to land use change (Peng et al. 2023). Our more robust analyses support a previous study demonstrating a forecasted decline in potential striped hyena habitat in Nepal (Bhandari et al. 2022). The effects of climate and land use change can result in species shifting to higher elevations and latitudes (Inman et al. 2016; Parmesan 2006), consistent with our findings as potential habitat of striped hyenas is projected to shift northward. Climate and land use change, along with anthropogenic mortality, superstitions, and traditional medicine practices, remain important threats to striped hyenas (AbiSaid and Dloniak 2015; Regmi et al. 2022).

In Nepal, hyenas persist in mid‐hill and low‐mountain valleys adjoining forests and agricultural lands. Climate models suggest a slight increase in habitat suitability by 2050 under both moderate (SSP1‐2.6) and extreme (SSP5‐8.5) scenarios, driven by moderate warming and vegetation shifts that create open, prey‐supporting habitats. By 2070, trends diverge under SSP1‐2.6; suitability declines as ecosystems stabilize into denser forests, while under SSP5‐8.5, intensified warming expands open and fragmented landscapes, increasing potential habitats (Bhandari et al. 2022). These findings underscore hyenas' dependence on ecotonal habitats and adaptability to modified landscapes, highlighting the need for conservation strategies that integrate climate projections with land‐use planning.

## 5 Conclusions

Warmer temperatures and increasingly dynamic precipitation are expected to positively influence striped hyena distribution, but overall, potential habitat will likely decline. This study highlights the need for proactive conservation measures and recommends raising local and national awareness of the importance of conserving striped hyenas and their habitat, including consideration of compensation policies to reduce human‐hyena conflicts. There is an urgent need for collaboration between government authorities and local people to collectively mitigate likely impacts of climate change and anthropogenic activities on striped hyena habitat.

Nearly 80% of suitable habitat for striped hyenas occurred outside protected areas, demonstrating an urgent need to increase conservation efforts beyond protected areas. Climate change could reduce habitats more than 80% by 2070, demonstrating the need for climate adaptation strategies. Land use, particularly urbanization and agriculture, also threatens striped hyena habitats, which will require conservation‐friendly policies. Finally, conservation efforts should prioritize regions outside protected zones by partnering with local communities, schools, and organizations. These programs should focus on raising awareness and integrating educational initiatives that highlight the importance of striped hyenas in maintaining ecosystem balance and controlling prey populations. Involving local communities, along with continued research and monitoring hyena distributions, is key to effective conservation. Incorporating this knowledge in national policies could improve efforts to conserve striped hyenas in Nepal.

## Author Contributions

**Surya Devkota:** data curation (equal), formal analysis (equal), investigation (equal), methodology (equal), writing – original draft (equal), writing – review and editing (equal). **Bashu Dev Baral:** investigation (equal), methodology (equal), writing – review and editing (equal). **Sandeep Regmi:** investigation (equal), methodology (equal), writing – original draft (equal), writing – review and editing (equal). **Bishnu Prasad Bhattarai:** funding acquisition (equal), investigation (equal), methodology (equal), writing – review and editing (equal). **Shivish Bhandari:** investigation (equal), methodology (equal), writing – review and editing (equal). **Hem Bahadur Katuwal:** funding acquisition (equal), investigation (equal), writing – review and editing (equal). **Basudha Rawal:** writing – review and editing (equal). **Jerrold L. Belant:** writing – review and editing (equal). **Rachana Shah:** writing – review and editing (equal). **Chiranjibi Prasad Pokheral:** writing – review and editing (equal). **Naresh Subedi:** writing – review and editing (equal). **Hari Prasad Sharma:** conceptualization (equal), data curation (equal), formal analysis (equal), funding acquisition (equal), investigation (equal), methodology (equal), project administration (equal), resources (equal), supervision (lead), validation (equal), visualization (equal), writing – original draft (equal), writing – review and editing (equal).

## Ethics Statement

Department of Forest and Soil Conservation (Permission ID: 476), Department of National Parks and Wildlife Conservation (Permission ID: 1165) permit for camera installation and data collection of wild mammal species for scientific purposes. In this survey, we did not handle/harm the animals. We identified the individual of Striped hyena after camera traps were removed from the field. We did not collect the specimens of any mammal species during this study.

## Conflicts of Interest

The authors declare no conflicts of interest.

## Supporting information


**Table S1:** Bioclimatic variables used for modeling of striped hyena.
**Figure S1:** Spearman's pairwise correlation coefficients between predictive variables in the striped hyena model. Annual mean temperature (bio1), mean diurnal range (bio2), isothermality (bio3), temperature seasonality (bio4), max temperature of warmest month (bio5), min temperature of coldest month (bio6), temperature annual range (bio7), mean temperature of wettest quarter (bio8), mean temperature of driest quarter (bio9), mean temperature of warmest quarter (bio10), mean temperature of driest quarter (bio11), annual precipitation (bio12), precipitation of wettest month (bio13), precipitation of driest month (bio14), precipitation seasonality (coefficient of variation) (bio15), precipitation of wettest quarter (bio16), precipitation of driest quarter (bio17), precipitation of warmest quarter (bio18), precipitation of coldest quarter (bio19).
**Figure S2:** Current potential habitat of striped hyena including in settlement.
**Figure S3:** Current and future potential habitat of striped hyena including in settlement too.

## Data Availability

Data are deposited at dryad https://doi.org/10.5061/dryad.hqbzkh1s1.
