# Supplementary material for: Current and Future Distribution of Striped Hyena in Nepal
Source: Ecol Evol. 2025 Sep 17;15(9):e72167. doi: 10.1002/ece3.72167 (PMC12443612; doi:10.1002/ece3.72167)
Supplement: Supplementary file 1 — Table S1: Bioclimatic variables used for modeling of striped hyena. Figure S1: Spearman's pairwise correlation coefficients between predictive variables in the striped hyena model. Annual mean temperature (bio1), mean diurnal range (bio2), isothermality (bio3), temperature seasonality (bio4), max temperature of warmest month (bio5), min temperature of coldest month (bio6), temperature annual range (bio7), mean temperature of wettest quarter (bio8), mean temperature of driest quarter (bio9), mean temperature of warmest quarter (bio10), mean temperature of driest quarter (bio11), annual precipitation (bio12), precipitation of wettest month (bio13), precipitation of driest month (bio14), precipitation seasonality (coefficient of variation) (bio15), precipitation of wettest quarter (bio16), precipitation of driest quarter (bio17), precipitation of warmest quarter (bio18), precipitation of coldest quarter (bio19). Figure S2: Current potential habitat of striped hyena including in settlement. Figure S3: Current and future potential habitat of striped hyena including in settlement too. [file ECE3-15-e72167-s001.docx]

# **Supporting Information**

**Supplementary Table 1**. Bioclimatic variables used for modeling of striped hyaena.

| Variables Name | Code |
| --- | --- |
| Annual Mean Temperature | bio1 |
| Mean Diurnal Range (Mean of monthly (max temp - min temp)) | bio2 |
| Isothermality (bio2/bio7) (×100) | bio3 |
| Temperature Seasonality (standard deviation ×100) | bio4 |
| Max Temperature of Warmest Month | bio5 |
| Min Temperature of Coldest Month | bio6 |
| Temperature Annual Range (bio5-bio6) | bio7 |
| Mean Temperature of Wettest Quarter | bio8 |
| Mean Temperature of Driest Quarter | bio9 |
| Mean Temperature of Warmest Quarter | bio10 |
| Mean Temperature of Coldest Quarter | bio11 |
| Annual Precipitation | bio12 |
| Precipitation of Wettest Month | bio13 |
| Precipitation of Driest Month | bio14 |
| Precipitation Seasonality (Coefficient of Variation) | bio15 |
| Precipitation of Wettest Quarter | bio16 |
| Precipitation of Driest Quarter | bio17 |
| Precipitation of Warmest Quarter | bio18 |
| Precipitation of Coldest Quarter | bio19 |


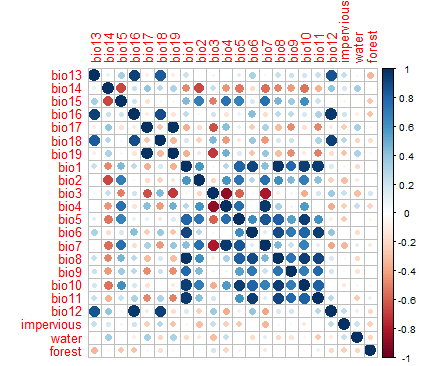


Supplementary Figure 1. Spearman pairwise correlation coefficients between predictive variables in the striped hyaena model. Annual mean temperature (bio1), Mean diurnal range ((bio2), Isothermality (bio3), temperature seasonality (bio4), max temperature of warmest month (bio5), min temperature of coldest month (bio6), temperature annual range (bio7), mean temperature of wettest quarter (bio8), mean temperature of driest quarter (bio9), mean temperature of warmest quarter (bio10), mean temperature of driest quarter (bio11), annual precipitation (bio12), precipitation of wettest month (bio13), precipitation of driest month (bio14), precipitation seasonality (coefficient of variation) (bio15), precipitation of wettest quarter (bio16), precipitation of driest quarter (bio17), precipitation of warmest quarter (bio18), precipitation of coldest quarter (bio19).


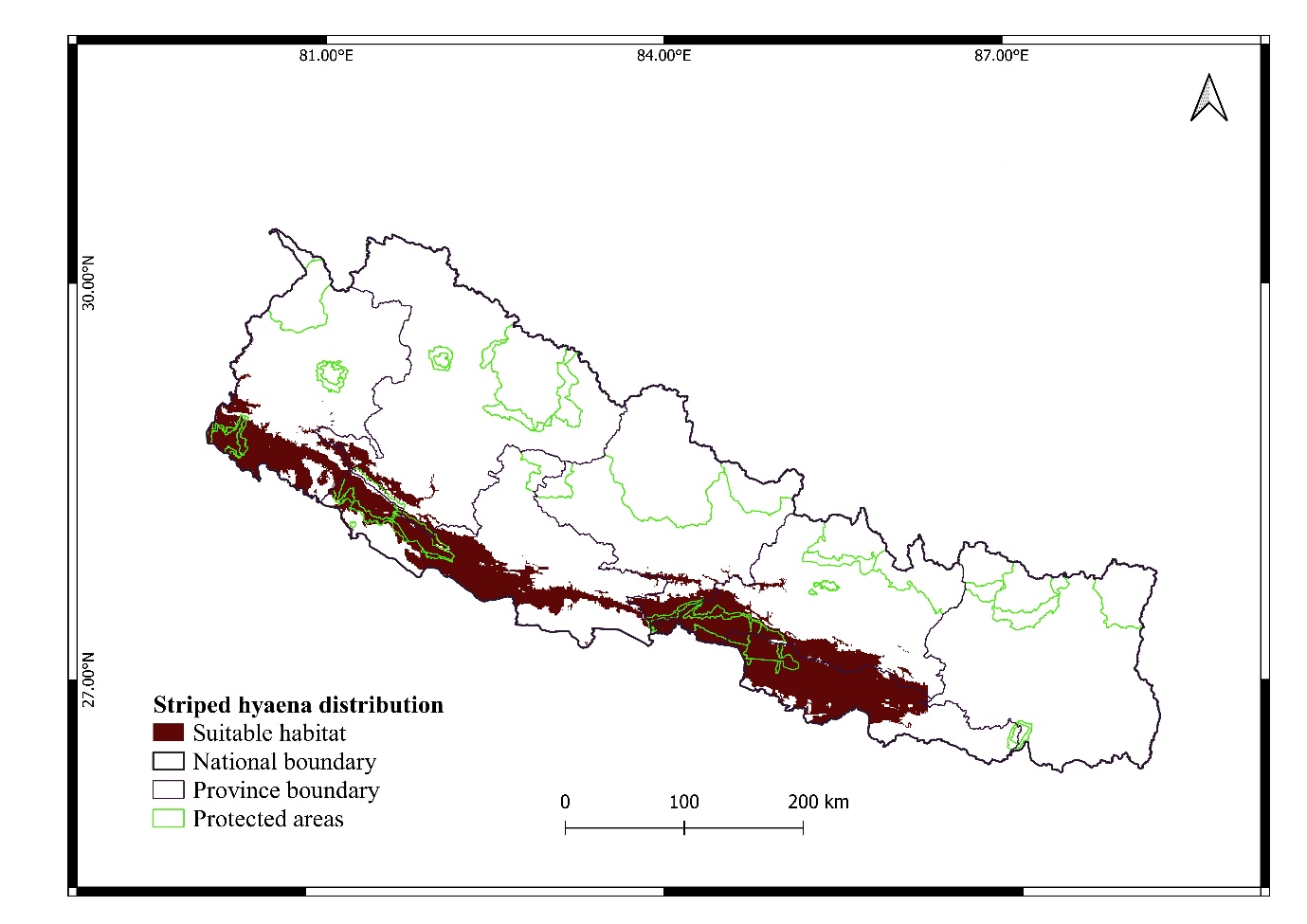


**Supplementary Figure 2.** Current potential habitat of striped hyaena including in settlement.


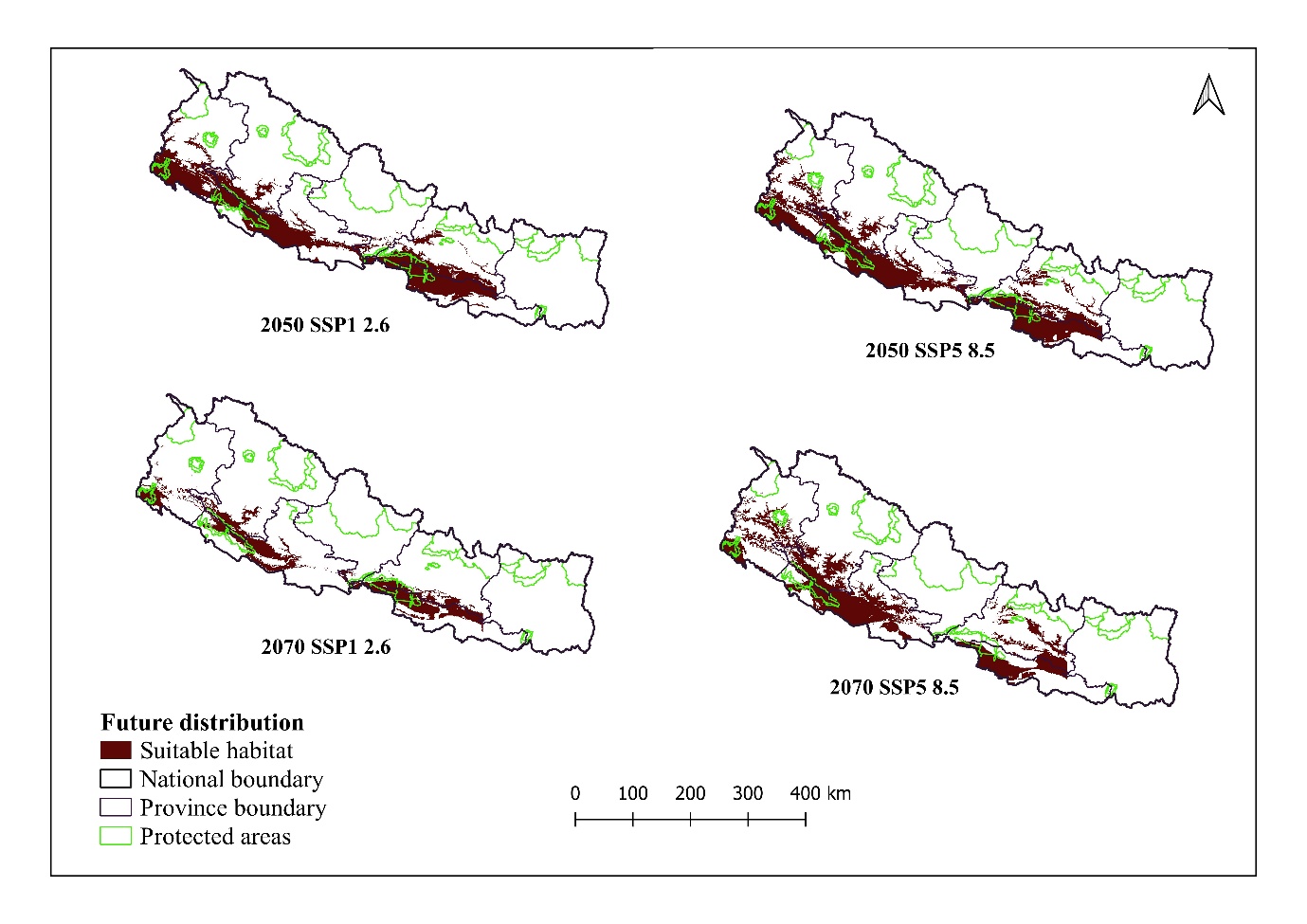


**Supplementary Figure 3.** Current and future potential habitat of striped hyaena including in settlement too.
